# Autoantibodies against MHC class I polypeptide-related sequence A are associated with increased risk of concomitant autoimmune diseases in celiac patients

**DOI:** 10.1186/1741-7015-12-34

**Published:** 2014-02-25

**Authors:** Antonio López-Vázquez, Lourdes Mozo, Rebeca Alonso-Arias, Beatriz Suárez-Álvarez, José Ramón Vidal-Castiñeira, Eduardo Arranz, Umberto Volta, Carlos Bousoño, Marcos López-Hoyos, Luís Rodrigo, Carlos López-Larrea

**Affiliations:** 1 Department of Immunology, Hospital Universitario Central de Asturias, Oviedo 33006, Spain; 2 Mucosal Immunology Laboratory, Department of Paediatrics and Immunology-IBGM, University of Valladolid, Valladolid, Spain; 3 Department of Gastroenterology and Internal Medicine, S. Orsola-Malpighi Hospital, University of Bologna, Bologna, Italy; 4 Department of Gastroenterology, Hospital Universitario Central de Asturias, Oviedo, Spain; 5 Department of Paediatrics, Hospital Universitario Central de Asturias, Oviedo, Spain; 6 Department of Immunology, Hospital Universitario Marqués de Valdecilla-IFIMAV, Santander, Spain; 7 Fundación Renal “Iñigo Álvarez de Toledo”, Madrid, Spain

**Keywords:** Autoantibodies, Autoimmune diseases, Celiac disease, MICA, NKG2D, Type 1 diabetes mellitus

## Abstract

**Background:**

Overexpression of autologous proteins can lead to the formation of autoantibodies and autoimmune diseases. MHC class I polypeptide-related sequence A (MICA) is highly expressed in the enterocytes of patients with celiac disease, which arises in response to gluten. The aim of this study was to investigate anti-MICA antibody formation in patients with celiac disease and its association with other autoimmune processes.

**Methods:**

We tested serum samples from 383 patients with celiac disease, obtained before they took up a gluten-free diet, 428 patients with diverse autoimmune diseases, and 200 controls for anti-MICA antibodies. All samples were also tested for anti-endomysium and anti-transglutaminase antibodies.

**Results:**

Antibodies against MICA were detected in samples from 41.7% of patients with celiac disease but in only 3.5% of those from controls (*P* <0.0001) and 8.2% from patients with autoimmune disease (*P* <0.0001). These antibodies disappeared after the instauration of a gluten-free diet. Anti-MICA antibodies were significantly prevalent in younger patients (*P* <0.01). Fifty-eight patients with celiac disease (15.1%) presented a concomitant autoimmune disease. Anti-MICA-positive patients had a higher risk of autoimmune disease than MICA antibody-negative patients (*P* <0.0001; odds ratio = 6.11). The risk was even higher when we also controlled for age (odds ratio = 11.69). Finally, we found that the associated risk of developing additional autoimmune diseases was 16 and 10 times as high in pediatric patients and adults with anti-MICA, respectively, as in those without.

**Conclusions:**

The development of anti-MICA antibodies could be related to a gluten-containing diet, and seems to be involved in the development of autoimmune diseases in patients with celiac disease, especially younger ones.

## Background

Celiac disease (CD) was previously considered a relatively rare pathology that appeared only in childhood, but is now recognized as being a very common disease that can be diagnosed at any age [1,2]. Its most typical characteristics are a strong genetic association with the human leukocyte antigen (HLA) alleles DQ2 and DQ8 [3,4] and its triggering by an environmental factor, the intake of gluten. The soluble fraction of gliadin has been identified as the cause of this intolerance, but many other gluten proteins can be toxic in CD [5]. These proteins induce an inflammatory process in the intestine of susceptible people, but inflammation regresses after elimination of gluten-containing foods from the diet, leading to the recovery of the structure and function of the mucosa [3].

Autoantibodies, especially those directed against the tissue transglutaminase (tTG) enzyme, commonly appear in CD [6]. These antibodies are very important in the diagnosis of CD, but their role in the pathogenesis of the disease remains controversial [7]. Several studies have suggested that these antibodies are directly involved in the pathogenesis of CD. Zanoni *et al*. demonstrated the role of anti-TG2 antibodies in gut mucosal damage in patients with CD [8]. Their study showed that these antibodies are able to recognize an epitope that is common to TG2 and the Toll-like receptor 4. This interaction leads to the activation of the Toll-like receptor 4 pathway, an important initiator of innate immunity. Other studies have shown that anti-tTG antibodies may play an important role in epithelial cell proliferation [9] and interfere with intestinal epithelial cell adhesion [10]. Additionally, these autoantibodies disturb angiogenesis and modulate vascular permeability *in vitro*[11,12]. Taken together, these observations suggest that future approaches to the study of CD should take the role of humoral immunity into account.

Other mechanisms, such as the MHC class I polypeptide-related sequence A (MICA)- Natural killer group 2, member D (NKG2D) interaction, are directly involved in the pathogenesis of the disease [13]. MICA and MICB show homology with classical HLA-class I, but have no role in antigen presentation. MICA and MICB are cell surface glycoproteins that are constitutively expressed in enterocytes [14]. These proteins are ligands for the killer cell lectin-like receptor subfamily K member 1, also known as NKG2D, which is an activating receptor that is mainly expressed in natural killer, CD8+ and γδT cells [15]. The MICA-NKG2D interaction in natural killer cells induces their cytolytic capacity, while in CD8+ T cells it acts as a co-stimulatory signal and complements antigen recognition by the T cell receptor [16]. MICA is strongly expressed in the enterocytes of patients with CD in response to the indirect toxic effect of gluten [17]. Furthermore, MICA binds the NKG2D receptor expressed on CD8+ intraepithelial lymphocytes and activates these T cells. This activation causes damage to the enterocytes, and could be the initiating event that ultimately leads to villous atrophy.

The tissue damage and increased expression of MICA may also induce the development of antibodies against this molecule. In fact, anti-MICA autoantibodies have been described in early-onset systemic lupus erythematosus (SLE) [18]. Moreover, these antibodies have been implicated in organ rejection in patients with renal [19-21] and cardiac transplants [22,23].

To test the hypothesis that the CD-related changes in gut mucosa may be associated with the development of antibodies against MICA, we investigated the presence of these antibodies in sera obtained from patients with active CD. We also considered the possibility that these antibodies play a role in the development of the additional autoimmune diseases (ADs) customarily associated with CD [24].

## Methods

### Study participants

A group of 383 patients diagnosed with CD (241 females, 142 males; mean age at diagnosis 22 ±21.96 years) by the Gastroenterology and Paediatric Departments of two Spanish and one Italian hospital between 2002 and 2012 were selected for this study. The diagnosis of CD was made in accordance with the revised criteria of the European Society for Paediatric Gastroenterology, Hepatology and Nutrition [25,26] and World Gastroenterology Organisation guidelines [27,28] In addition to clinical features, all patients were positive for anti- tTG and/or anti-endomysium antibodies. They also presented with a variable degree of intestinal mucosal damage (Marsh I to Marsh IIIc), before starting a gluten-free diet (GFD). A second serum sample was obtained from all patients after at least one year on a GFD. These samples were analyzed to establish treatment compliance and to assess the influence of a GFD on titers of anti-MICA antibodies.

All patients were typed for HLA-DQA1* and HLA-DQB1* alleles. Similar to the prevalence noted in Caucasian populations [29], 88% of the patients were HLA-DQ2+ and 11% were HLA-DQ8+. The clinical features of the patients are illustrated in Table 1. Additionally, the various ADs detected in these patients are listed in Table 2.

**Table 1:** Clinical and analytical features of patients and healthy controls

	**Patients with celiac disease**	**Patients with autoimmune disease**	**Healthy controls n = 200**
	**n = 383**	**n = 428**	
Gender (female/male)	241/142	280/148	112/88
Mean age (SD)	22 (21.76)	39 (23.04)	23 (18.74)
Biopsy			
Marsh I	7 (1.8%)	-	-
Marsh II	32 (8.3%)	-	-
Marsh IIIa	63 (16.4%)	-	-
Marsh IIIb	70 (18.3%)	-	-
Marsh IIIc	211 (55.1%)	-	-
HLA-DQ2-positive^a^	338 (89.2%)^b^	182 (42%)	44 (22%)
HLA-DQ8-positive^a^	42 (11.0%)	152 (35%)	18 (9%)
HLA-DQ2 and DQ8-negative	14 (3.6%)	136 (31.8%)	138 (69%)
Autoimmune diseases	58 (15.1%)	428 (100%)	-

^a^Includes DQ2-DQ8-positive patients; ^b^*P* <0000.1; odds ratio = 24.19; 95% confidence interval = 15.815, 36.997.

**Table 2:** Autoimmune diseases in patients with and without celiac disease

	**With celiac disease**	**Without celiac disease**
	**n = 58**	**n = 428**
Type 1 diabetes	22 (37.9%)	106 (24.8%)
Autoimmune thyroiditis	9 (15.5%)	47 (11.0%)
Systemic lupus erythematosus	7 (12.1%)	92 (21.5%)
Rheumatoid arthritis	6 (10.3%)	51 (11.9%)
Pernicious anemia	3 (5.2%)	22 (5.1%)
Primary biliary cirrhosis	3 (5.2%)	17 (4.0%)
Juvenile idiopathic arthritis	1 (1.7%)	4 (1.0%)
Autoimmune hepatitis	3 (5.2%)	15 (3.5%)
Crohn’s disease	2 (3.4%)	40 (9.3%)
Ulcerative colitis	-	9 (2.1%)
Mixed connective tissue disease	1 (1.7%)	1 (0.2%)
Addison’s disease	-	3 (0.7%)
Sjögren’s syndrome	1 (1.7%)	21 (4.9%)

Another group of 428 patients (mean age 39 ± 3.04 years, 65.4% females) diagnosed for several ADs but without CD were selected to establish whether the presence of anti-MICA antibodies is related to CD or if they are another serological marker of autoimmunity. The composition of this group with respect to represented ADs is similar to that for patients with CD and ADs (Table 2).

Finally, 200 healthy individuals matched by age (mean age 23 ±18.74 years, 54% females) were selected for comparison to patients with CD. Control individuals had no history of intolerance to gluten or derivatives, iron deficiency, anemia, or abnormalities in biochemical studies, and were negative for anti-TG antibodies. To match healthy controls and patients with CD by age, the proportion of individuals aged ≤14 years was similar in both groups (47% versus 52%). Children used as healthy controls were selected from those with suspected allergic reactions who were studied in the Allergology Department of Hospital Universitario Central de Asturias.

The study was approved by the Ethics Committee of each hospital (Regional Ethics Committee of Clinical Research of Principado de Asturias; Ethical Committee of S. Orsola Malpighi Hospital, University of Bologna; Ethical Committee of Clinical Research from the Clinic Hospital, University of Valladolid; Cantabria Ethics Committee for Biomedical Research). All patients and controls or their parents gave written informed consent.

### Determining anti-endomysium and anti-transglutaminase autoantibodies

Anti-endomysium antibodies were detected by indirect immunofluorescence using tissue sections from monkey esophagus (Biosystems, Barcelona, Spain). A titer of 1:10 or more was considered positive. Anti-tTG autoantibodies were detected using an ELISA kit from Orgentec (Mannheim, Germany). A value greater than 10 units was considered positive. Patients’ sera were tested for total immunoglobulins (Ig) to detect IgA deficiency. In participants with IgA deficiencies, IgG equivalents of the above tests were used.

### HLA and MICA typing

Genomic DNA from all patients was isolated and typed for the HLA-DQ allele using DNA PCR amplification with sequence-specific primers with the PROTRANS^TM^ Domino System HLA Celiac Disease kit (Protrans, Ketsch, Germany). Additionally, MICA gene polymorphisms from 100 patients were typed with a LABType® SSO MICA typing kit (One-Lambda, Los Angeles, CA, USA). All determinations were done in accordance with the manufacturers’ protocols.

### Anti-MICA antibody detection

MICA antibodies were identified by LABScreen® assays (One-Lambda) using Luminex xMAP technology (Luminex Corp., Austin, TX, USA), following the manufacturer’s specifications. Serum samples from patient with CD and controls were tested against MICA alleles using the LABScreen® Mixed kit for general screening. Positive sera were re-tested using LABScreen® MICA Single Antigen to measure the specificity of the antibodies. The fluorescent signal for each MICA allele-coated bead was measured using LABScan 100^TM^ Flow Cytometry and analyzed by HLA-Fusion^TM^ software (One-Lambda). Antibodies against MICA alleles were considered positive when the mean fluorescent intensity (MFI) of each bead was above a cut-off value of 500 in LABScreen® Mixed and 2000 in LABScreen® MICA Single Antigen, as suggested by the manufacturer. In all cases, anti-MICA antibodies detected by this technique were of the IgG isotype.

### Statistics

Descriptive analyses were used to characterize the study population. The chi-square contingency test was used to compare dichotomous variables and the unpaired t-test was used to compare group differences of continuous variables. Multivariate logistic regression was used to model the variables that were significant in univariate analyses or that were of clinical relevance. All analyses were done using SPSS v.15.0. Values of *P* <0.05 were considered significant in all cases.

## Results

### Anti-MICA autoantibodies are more prevalent in patients with celiac disease

Our initial aim was to analyze anti-MICA antibodies in sera obtained from patients diagnosed with CD and from healthy controls. We found that their presence was clearly associated with CD. Anti-MICA antibodies were detected in 159 of 383 patients with CD (41.5%) compared with 3.5% of the healthy controls (*P* <0.0001; Table 3). In other words, the odds of individuals with CD presenting anti-MICA antibodies were 19 times those of healthy controls. Next, to establish whether anti-MICA autoantibodies are a characteristic of CD but not a frequent feature of other ADs, we compared their frequency in patients with CD with the group who were diagnosed only with ADs. Our results demonstrated that these autoantibodies were associated with CD (41.5% in the CD group versus 8.2% in the AD group; *P* <0.0001; odds ratio = 7.97; 95% confidence interval: 5.38, 11.90). Anti-MICA antibodies were no longer present in the additional sample of 75% of the patients with CD who had been on a GFD for at least one year. The second serum was positive for anti-tTG antibodies in ten patients, six of whom had anti-MICA antibodies (data not shown).

**Table 3:** Prevalence of anti-MICA autoantibodies in patients and healthy controls

**Anti-MICA**	**Patients with celiac disease**	**Patients with autoimmune disease**	**Healthy controls**
	**n = 383**	**n = 428**	**n = 200**
Positive	159 (41.5%)	35 (8.2%)^a^	7 (3.5%)^b^
Negative	224 (58.5%)	393 (91.8%)	193 (96.5%)

^a^*P* <0.0001; odds ratio = 7.97; 95% CI = 5.38, 11.90. ^b^*P* <0.0001; odds ratio = 19.57; 95% confidence interval = 8.96, 42.74.

We compared the maximum MFI of anti-MICA autoantibodies with the values of anti-tTG ones, but found no correlation between them (Additional file 1: Figure S1). The distribution of maximum MFI among the different groups of patients was also analysed, but revealed no statistically significant differences (Additional file 1: Figure S2).

The specificities of the anti-MICA antibodies were determined in 50 randomly selected patients, combining Luminex single antigen analysis with MICA genotyping. In all cases, the antibodies recognized self-MICA alleles. Moreover, 22 patients also developed antibodies against other MICA variants. The most frequent MICA antigen detected was MICA*027, which corresponds to the MICA A5.1 transmembrane polymorphism, which has previously been associated with CD [29-31]. The allele was present in 74% of patients (data not shown).

### Anti-MICA autoantibodies are related to age at diagnosis

Having identified the presence of anti-MICA antibodies, we investigated whether other factors related to CD had influenced their induction. First, we analyzed the influence of patient age at diagnosis on the development of antibodies (Table 4). Clearly, anti-MICA autoantibodies were more prevalent at early ages: the mean age of people positive for anti-MICA was 21.03 years compared to 31.60 years for people negative for anti-MICA; and the median age was significantly lower in positive compared to negative individuals (12 versus 31 years; *P* <0.01). The tendency of antibodies to appear at a younger age was apparent in all patient and control groups (Table 4).

**Table 4:** Distribution of anti-MICA autoantibodies in different patient groups included in the study by age at diagnosis

**Groups**	**Anti-MICA**	**n**	**Mean**	**SD**	**Median**	***P***
All	Negative	810	31.60	23.14	31	-
	Positive	201	21.03	21.89	12	< 0.01
Healthy controls	Negative	193	22.75	18.390	19	-
	Positive	7	41.86	20.408	54	-
Patients with celiac disease	Negative	224	24.85	22.320	23	-
	Positive	159	17.74	20.309	7	-
Patients with autoimmune disease	Negative	393	39.81	22.88	41	-
	Positive	35	31.86	23.85	26	-

No relationship was found between the presence of anti-MICA antibodies and gender or the degree of the Marsh lesion (see Additional file 1: Tables S1 and S2).

### The risk of developing additional autoimmune diseases in patients with celiac disease is associated with anti-MICA autoantibodies

Patients with CD had a higher incidence of additional ADs, mainly type 1 diabetes [22,24,32]. The prevalence of these diseases was relatively high in our population; 58 patients with CD (15.1%) were found to have concomitant disease (Table 1). To identify possible risk factors related to CD that could be involved in the development of these pathologies, a multivariate analysis was performed. This indicated that gender, HLA-DQ and Marsh type were not associated with the presence of ADs in these patients. However, patients with an additional autoimmune pathology were older, on average, than those who were affected by CD alone (mean age, 36 ±20.18 versus 18 ±20.62 years, *P* <0.001; data not shown).

We investigated the possible influence of anti-MICA autoantibodies on the development of additional ADs (Table 5). The majority of patients with CD and ADs were positive for anti-MICA antibodies (79.3%; 46 out of 58), whereas only 34.8% (113 out of 325) of patients affected by CD alone had anti-MICA (Table 5), demonstrating that the development of ADs in patients with CD was clearly associated with the presence of anti-MICA autoantibodies (*P* <0.0001; odds ratio = 6.11; 95% confidence interval: 3.22, 11.59). When the analysis also adjusted for age, the risk associated with anti-MICA was notably higher (*P* <0.0001; odds ratio = 11.69; 95% confidence interval: 5.49, 24.90).

**Table 5:** Risk of developing concomitant autoimmune diseases in patients with celiac disease with respect to the presence of anti-MICA autoantibodies

		**Celiac disease only**	**Celiac disease with autoimmune disease**
		**n = 325**	**n = 58**
Anti-MICA-positive		113 (34.8%)	46 (79.3%)
Anti-MICA-negative		212 (65.2%)	12 (20.7%)
	***P***	**Odds ratio**	**95% confidence interval**
Anti-MICA	<0.0001	6.11	3.22, 11.59
Anti-MICA^a^	<0.0001	11.69	5.49, 24.90

Risk associated with the presence of anti-MICA in all patients with CD. ^a^Adjusted by age at diagnosis.

As previously mentioned, ADs were more prevalent in patients diagnosed with CD as adults, whereas anti-MICA autoantibodies were more frequent in those diagnosed with CD at pediatric age. Given the well-established influence of age at diagnosis on the risk of developing concomitant ADs due to anti-MICA, we decided to investigate the influence of these autoantibodies in pediatric and adult patients. The distribution in the two groups indicated that autoantibodies were present in 74.4% of adult patients with concomitant ADs, with an associated risk of 10.03 (*P* <0.0001), whereas 93.3% of pediatric patients with CD and an additional autoimmune pathology had anti-MICA antibodies (*P* <0.01). The risk associated with anti-MICA in children was 16.28, which was higher than that in adult patients (Table 6).

**Table 6:** Risk of developing concomitant autoimmune diseases in patients with celiac disease with respect to the presence of anti-MICA autoantibodies, according to age at diagnosis

	**Celiac disease only**		**Celiac disease with autoimmune diseases**	
**Age groups**	**Pediatric**	**Adult**	**Pediatric**	**Adult**
	**(n = 186)**	**(n = 139)**	**(n = 15)**	**(n = 43)**
Anti-MICA-positive	76 (40.9%)	26 (18.7%)	14 (93.3%)	32 (74.4%)
Anti-MICA-negative	110 (59.1%)	113 (81.3%)	1 (6.7%)	11 (25.6%)
Anti-MICA		***P***	**Odds ratio**	**95% confidence interval**
Pediatric		<0.01	16.28	2.10, 126.34
Adult		<0.0001	10.03	4.64, 21.66

Risk associated with the presence of anti-MICA in pediatric (≤14 years) and adult (>14 years) groups.

## Discussion

The mechanisms leading to systemic autoimmune aggression in patients with CD remain unknown. NKG2D and their ligands could play a significant role in the development of autoimmunity. In fact, these molecules are involved in the pathogenesis of some ADs that are commonly associated with CD, such as type 1 diabetes and rheumatoid arthritis. For example, blockage of NKG2D in the pre-diabetic stage in non-obese diabetic mice prevents the development of diabetes [33]. In rheumatoid arthritis, MICA and MICB are aberrantly expressed in pathological tissue from affected joints and could be involved in the continuation of the autoreactive process [34].

We found anti-MICA antibodies to be present in half of the patients with untreated CD but in only 3.5% of healthy controls. Moreover, anti-MICA antibodies were present in most patients diagnosed with CD plus other ADs, but were rare in patients solely with ADs. Furthermore, anti-MICA antibodies disappeared from most patients after a year on a GFD, similar to what occurs with anti-tTG antibodies. These results imply that, generally, the relationship between anti-MICA and associated ADs is directly linked to CD rather than being a phenomenon specifically associated with autoimmunity. However, as previously mentioned, early-onset SLE may be an exception to this pattern, since these antibodies have also been described in this disease [18].

Previous studies of CD show that MICA protein is overexpressed in enterocytes obtained from the damaged mucosa of patients [13,17]. A high level of MICA expression seems to promote mucosal damage by infiltrating intraepithelial CD8+ T lymphocytes, which express the MICA receptor, NKG2D. In fact, other authors have suggested that overexpression of MICA could be an initiating event in the progression of mucosal damage [35-37]. In the early phase of CD, some gluten-derived peptides, which have been characterized as non-immunogenic, are directly involved in increasing expression of MICA and IL-15. These ‘toxic’ peptides activate the innate immunity through an unknown pathway, leading to the destruction of the gut mucosa [35]. As has been demonstrated in other inflammatory processes, massive cellular destruction may be involved in the development of autoantibodies [38]. In CD, an initially higher level of expression of MICA due to gluten-derived peptides followed by the destruction of enterocytes by intraepithelial lymphocytes could trigger the subsequent development of anti-MICA antibodies in susceptible individuals.

The fact that fewer than half of patients with CD have anti-MICA prevents the use of this antibody as a diagnostic marker. However, the greater prevalence of these autoantibodies in patients with additional ADs observed in our study implies that anti-MICA antibodies might be useful for predicting the risk of AD development. ADs are diseases that are frequently associated with CD. Several studies have found a close association between a long period of gluten exposure [15,39] and a common genetic background, although others found no relationship between ADs and prolonged gluten intake in CD [40]. We found that ADs in our group of patients were more frequent in adults than in the young, but this could be related to the established effect of age on the prevalence of these diseases, rather than due to the duration of gluten exposure. Moreover, the association of anti-MICA autoantibodies with the presence of additional ADs is substantially modified by age. Surprisingly, although ADs are more frequent in adults with CD, the risk associated with anti-MICA antibodies of developing concomitant AD is clearly greater in childhood, so the determination of these autoantibodies may be very useful in clinical practice to establish the risk in children. In our study, only one pediatric patient with ADs was negative for anti-MICA autoantibodies. In children, CD and other ADs are usually more aggressive processes than in adults. In CD, this could result in increased expression of MIC molecules in younger patients, which would favor the generation of autoantibodies. The presence of anti-MICA autoantibodies could augment tissue destruction and lead to the early development of ADs. As mentioned previously, Dai *et al*. also demonstrated the presence of anti-MICA autoantibodies in 27 patients with juvenile-onset SLE [18]. Our group of 428 patients with AD included 92 diagnosed with SLE. Six of these patients were classified as juvenile-onset SLE and all had anti-MICA (data not shown). Although these antibodies have not been directly implicated in the disease, they might be an additional risk factor in the development of SLE and an interesting biomarker in the diagnostic of this disease in childhood.

### Limitations

Anti-MICA autoantibodies were present in fewer than half of patients with CD, which make them less useful than other autoantibodies, such as anti-tTG or anti-deamidated gliadin, as a diagnostic biomarker. Moreover, all patients included in the study were positive for anti-tTG although the absence of these autoantibodies does not discard the diagnosis of CD, especially in children. Therefore, it would be of great interest to analyze anti-MICA in a population of patients with CD patients who do not have anti-tTG autoantibodies.

Another limitation of this study is the small number of patients with CD and a concomitant AD, which is especially relevant in the group of pediatric patients.

## Conclusions

We have shown here that the development of anti-MICA antibodies is associated with a gluten-containing diet in patients with CD. In addition, anti-MICA antibodies are apparently related to the development of ADs, especially in younger patients although the number of individuals in this study group was small. The determination of these autoantibodies might be less suitable than others for the diagnosis of the disease, but it could be very useful in clinical practice for predicting the development of associated ADs in patients with CD.

The mechanism by which these antibodies could influence the development of autoimmunity might be related to direct tissue damage, the activation of cellular immunity, or another mechanism similar to that demonstrated for anti-tTG in other studies [8-12]. Further work is needed to establish the role and significance of these autoantibodies in CD and, possibly, in other ADs such as SLE, especially in patients with a young age of onset.

## Abbreviations

AD: autoimmune disease; CD: celiac disease; CI: confidence interval; ELISA: enzyme-linked immunosorbent assay; GFD: gluten-free diet; HLA: human leukocyte antigen; Ig: immunoglobulin; IL: interleukin; MFI: mean fluorescence intensity; MICA: MHC class I polypeptide-related sequence; NK: natural killer lymphocyte; PCR: polymerase chain reaction; SLE: systemic lupus erythematosus; tTG: tissue transglutaminase.

## Competing interests

The authors declare that they have no competing interests.

## Authors' contributions

ALV: study concept and design, analysis and interpretation of data and writing of manuscript. LM: acquisition of data, technical and material support. RAA: statistical analysis. BSÁ: acquisition of data, technical and material support. JRVC: acquisition of data, technical and material support. EA: acquisition of data, critical revision of the manuscript. UV: acquisition of data, critical revision of the manuscript. CB: acquisition of data, critical revision of the manuscript. MLH: acquisition of data, technical and material support. LR: acquisition of data, critical revision of the manuscript and study supervision. CLL: study concept and design, analysis and interpretation of data, writing of manuscript and study supervision. All authors read and approved the final version of the manuscript for submission.

## Pre-publication history

The pre-publication history for this paper can be accessed here:

http://www.biomedcentral.com/1741-7015/12/34/prepub

## Supplementary Material

Additional file 1: Figure S1 Titration curves of anti-tTG autoantibodies and anti-MICA autoantibodies in three representative patients with CD. **Figure S2.** Dot box plot representing the distribution of maximum MFI in the distinct groups of patients. No comparisons exhibited statistically significant differences between the groups. **Table S1.** (A) Distribution of MICA in patients with CD according to gender. (B) Distribution of genders in patients with CD according to the presence or absence of anti-MICA autoantibodies. **Table S2.** (A) Distribution of genders in patients with CD according to the presence or absence of anti-MICA autoantibodies. (B) Severity of mucosal lesion (Marsh classification) in patients with CD according to the presence or absence of anti-MICA autoantibodies. Click here for file
