# Supplementary material for: Autoantibodies against MHC class I polypeptide-related sequence A are associated with increased risk of concomitant autoimmune diseases in celiac patients
Source: BMC Med. 2014 Feb 25;12:34. doi: 10.1186/1741-7015-12-34 (PMC3945941; doi:10.1186/1741-7015-12-34)
Supplement: Additional file 1: Figure S1 — Titration curves of anti-tTG autoantibodies and anti-MICA autoantibodies in three representative patients with CD. Figure S2. Dot box plot representing the distribution of maximum MFI in the distinct groups of patients. No comparisons exhibited statistically significant differences between the groups. Table S1. (A) Distribution of MICA in patients with CD according to gender. (B) Distribution of genders in patients with CD according to the presence or absence of anti-MICA autoantibodies. Table S2. (A) Distribution of genders in patients with CD according to the presence or absence of anti-MICA autoantibodies. (B) Severity of mucosal lesion (Marsh classification) in patients with CD according to the presence or absence of anti-MICA autoantibodies. [file 1741-7015-12-34-S1.docx]

**Additional Figure 1.** Titration curves of anti-tissue transglutaminase autoantibodies and anti-MICA autoantibodies in three representative patients with coeliac disease.


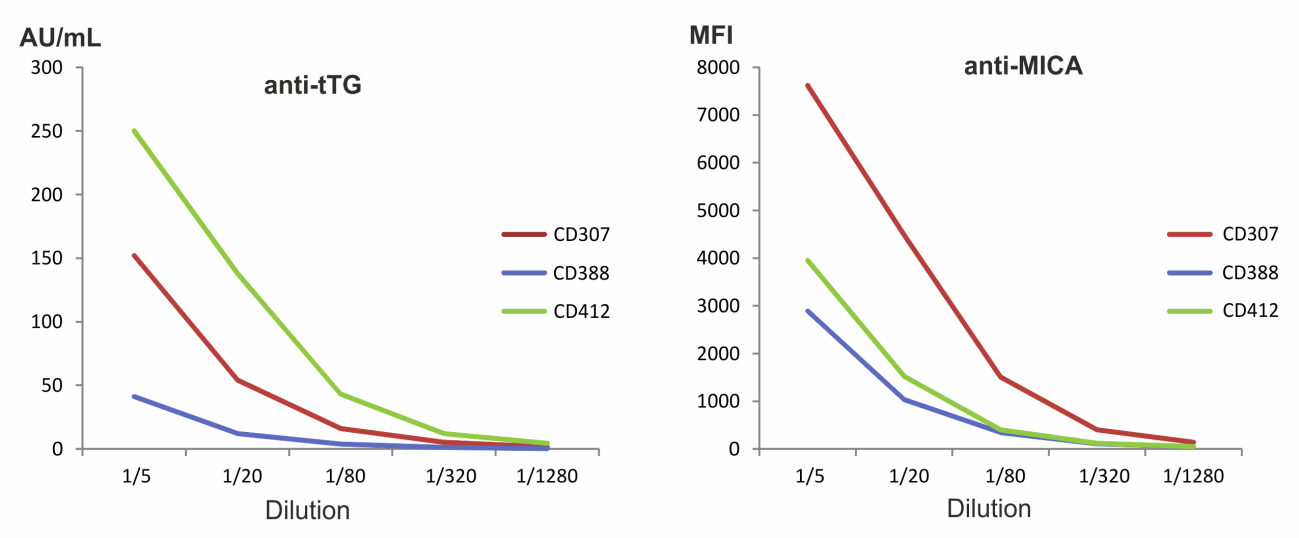


AU/mL: arbitrary units per millilitre.

MFI: Mean fluorescence intensity

**Additional Figure 2.** Dot box plot representing the distribution of maximum mean fluorescence intensity (MFI) in the distinct groups of patients. No comparisons exhibited statistically significant differences between the groups.





**Additional Table 1:**

A) Distribution of MICA in coeliac patients according with gender

|  | Female  n = 241 | Male  n = 142 |
| --- | --- | --- |
| Anti-MICA-negative | 148 (61.4%) | 76 (53.5%) |
| Anti-MICA-positive | 93 (38.6%) | 66 (46.5%) |

B) Distribution of genders in coeliac patients according to the presence or absence of anti-MICA autoantibodies.

|  | Female | Male |
| --- | --- | --- |
| Anti-MICA-negative  n = 224 | 146 (66.1%) | 33.9 (9.4%) |
| Anti-MICA-positive  n = 159 | 93 (58.5%) | 41.5 (6.9%) |

**Additional table 2:**

A) Distribution of MICA in coeliac patients according with Marsh classification

|  | MARSH I  n = 7 | MARSH II  n = 32 | MARSH IIIa  n = 63 | MARSH IIIb  n = 70 | MARSH IIIc  n = 211 |
| --- | --- | --- | --- | --- | --- |
| Anti-MICA-negative | 6 (85.7%) | 21 (65.6%) | 34 (54.0%) | 43 (61.4%) | 120 (56.9%) |
| Anti-MICA-positive | 1 (14.3%) | 11 (34.4%) | 29 (46.0%) | 27 (39.6%) | 91 (43.1%) |

B) Severity of mucosal lesion (Marsh classification) in coeliac patients according to the presence or absence of anti-MICA autoantibodies.

|  | MARSH I | MARSH II | MARSH IIIa | MARSH IIIb | MARSH IIIc |
| --- | --- | --- | --- | --- | --- |
| Anti-MICA-negative  n = 224 | 6 (2.7%) | 21 (9.4%) | 34 (15.2%) | 43 (19.2%) | 120 (53.6%) |
| Anti-MICA-positive  n = 159 | 1 (0.6%) | 11 (6.9%) | 29 (18.2%) | 27 (17.0%) | 91 (57.2%) |
